# *Rhodococcus fascians* infection after haematopoietic cell transplantation: not just a plant pathogen?

**DOI:** 10.1099/jmmcr.0.005025

**Published:** 2016-03-03

**Authors:** Melissa C. Austin, Teal S. Hallstrand, Daniel R. Hoogestraat, Gregory Balmforth, Karen Stephens, Susan Butler-Wu, Cecilia C. S. Yeung

**Affiliations:** ^1^​ Department of Pathology, Walter Reed National Military Medical Center, 8901 Rockville Pike, Bethesda, MD 20889, USA; ^2^​ Departments of Pulmonary and Critical Care Medicine, University of Washington, 1959 NE Pacific St, Seattle, WA 98105, USA; ^3^​ Department of Laboratory Medicine, University of Washington, 1959 NE Pacific St, Seattle, WA 98105, USA; ^4^​ Department of Radiology, Swedish Medical Center, 5300 Tallman Ave NW, Seattle, WA 98107, USA; ^5^​ Department of Anatomic Pathology, University of Washington, 1959 NE Pacific St, Seattle, WA 98105, USA; ^6^​ Fred Hutchinson Cancer Research Center, 1100 Fairview ave N, Mailstop G7-910, Seattle, WA 98109, USA

**Keywords:** Immunocompromised hosts, opportunistic infection, post-transplant complication, *Rhodococcus fascians*

## Abstract

**Introduction:**

*Rhodococcus* spp. have been implicated in a variety of infections in immunocompromised and immunocompetent hosts. *Rhodococcus equi* is responsible for the majority of reported cases, but *Rhodococcus erythropolis*, *Rhodococcusgordoniae* and *Rhodococcusruber* infections have been described. There are no prior reports of human infection with *Rhodococcus fascians*.

**Case presentation:**

We describe the unexpected finding of *R. fascians* in liver lesions incidentally noted at autopsy in an immunosuppressed patient status after bone-marrow transplant for acute lymphoblastic leukaemia who died of unrelated causes (septic shock due to *Clostridium difficile* colitis). At autopsy, an otherwise unremarkable liver contained several dozen well-demarcated sclerotic-appearing lesions measuring 0.1–0.3 cm in size. The absence of other bacterial or fungal DNA in the setting of histologically visible organisms argues against its presence as a contaminant and raises the consideration that *R. fascians* represents a human pathogen for the immunocompromised.

**Conclusion:**

Whether it represents the sole infectious agent responsible for the miliary lesions or a partially treated co-infection is impossible to determine, but our finding continues to reinforce the importance of molecular techniques in associating organisms with sites of infection and optimizing treatment of infectious diseases.

## Introduction

Rhodococci are a group of Gram-positive, non-motile, non-spore-forming, aerobic organisms that are typically considered benign (van der Geize & Dijkhuizen, 2004) but that have been implicated in a variety of infections in both immunocompromised and immunocompetent hosts. A member of the family *Nocardiaceae*, they are a fastidious group of organisms that are difficult to culture, and 16S rRNA gene studies have been shown to be a sensitive and specific method of detecting these organisms (Bharadwaj *et al.*, 2012). Several species of the genus *Rhodococcus* have been reported as a cause of human disease, with *Rhodococcus equi* responsible for the majority of infections, but *Rhodococcusruber*, *Rhodococcuserythropolis* and *Rhodococcus gordoniae* infections have also been described in humans. Rhodococci typically infect humans via inhalation, usually after aerosolization of the micro-organisms (such as occurs with soil turnover in farming), and they have the ability to form biofilms (Orr *et al.*, 2004).

*Rhodococcus fascians* has been described as a plant pathogen and is primarily responsible for leafy gall disease in a variety of plants (Goethals *et al.*, 2001). There are no prior reports of human infection, but *R. fascians* is known to aerosolize and form biofilms (Francis *et al.*, 2012) in a similar fashion to other *Rhodococcus* spp. that infect humans. They are pleomorphic bacteria that can form branched hyphae or can break apart into either rod- or cocci-shaped bacteria.

As medical practice gains sophistication, many treatment regimens such as chemotherapy, total body irradiation and transplantation cause immunosuppression. Immunocompromised patients are at risk for pathogens that normally do not cause infections in healthy individuals, and it is important to recognize this expanded range of possible pathogens in order to fully counsel patients on the necessary precautions during their treatment course. Beyond this, identification of these unexpected pathogens can aid pathologists and the clinical care team in recognizing and making correct infectious disease diagnoses, which ultimately helps guide clinicians in the proper management of these patients when they succumb to unusual and/or difficult-to-treat infections.

## Case report

Our patient was a 44-year-old woman who initially presented with an abnormal complete blood count prompting a bone-marrow biopsy, which revealed Philadelphia chromosome-positive B-cell acute lymphoblastic leukaemia. She received three cycles of hyper-CVAD (hyperfractionated chemotherapy regimen cyclophosphamide, vincristine, doxorubicin) with imatinib, rituximab and dasatinib to which she had an apparent complete and sustained response, but her pre-transplant course was complicated by multiple bacterial, fungal and viral opportunistic infections, primarily of the respiratory tract. She received non-myeloablative conditioning therapy consisting of fludarabine and low-dose total body irradiation followed by haematopoietic cell transplantation (HCT). Her post-transplant course was complicated by *Klebsiella oxytoca* bacteraemia, *Clostridium difficile* colitis, cytomegalovirus (CMV) reactivation, low-level human herpesvirus-6 viraemia, BK virus viruria/viraemia, renal insufficiency (presumably medication related), and rash and diarrhoea attributed to graft-versus-host-disease (GVHD).

The patient had no clinical or pathological evidence of residual/recurrent leukaemia, and she appeared to be improving from an infectious disease standpoint when she presented (the day before her death) acutely to our outpatient clinic with fever and diarrhoea for which she was promptly transferred to the Emergency Department. Her admission imaging studies showed severe colitis with mesenteric inflammation concerning for infection, and intravenous vancomycin, piperacillin-tazobactam and metronidazole were started. Initial laboratory results were significant for positive *C. difficile* PCR and rising CMV titres. However, during pre-operative assessment, she became hypoxic and hypotensive and, despite resuscitative efforts, died.

**Fig. 1:**
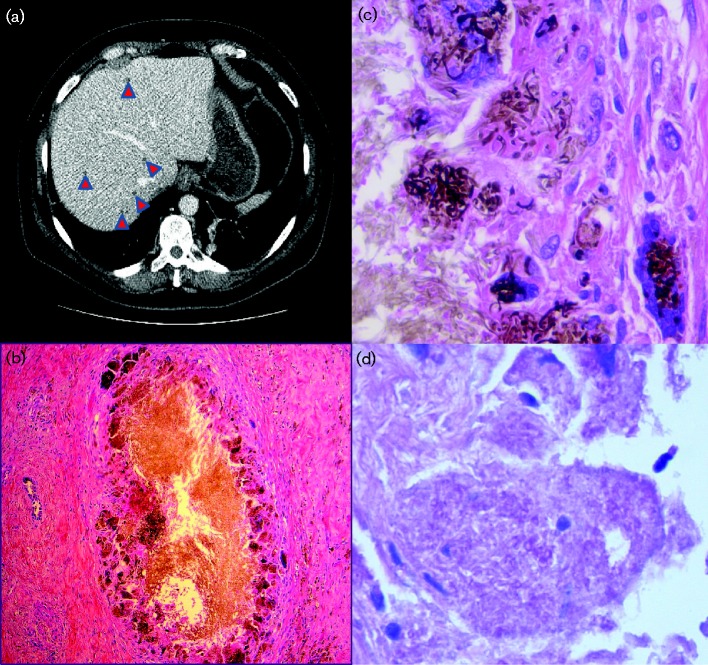
(a) CT of the abdomen showing scattered low-attenuation lesions (arrowheads). (b) Separate lesion containing filamentous organisms (methenamine silver stain with haematoxylin and eosin counterstain; magnification × 100). (c) Intracellular filamentous organisms (methenamine silver with haematoxylin and eosin counterstain; magnification × 1000). (d) Intracellular filamentous organisms (periodic acid–Schiff stain; magnification × 1000).

An autopsy was requested to determine the causative agent(s) of the patient's sepsis, to determine whether abdominal compartment syndrome was present, to evaluate the degree of GVHD and to document the extent of CMV infection. A thorough post-mortem review of the medical records revealed a history of multiple low-attenuation liver lesions seen on a computed tomography (CT) scan performed several months pre-transplant at an outside institution (Fig. 1a); treating physicians at the outside facility had initiated broad-spectrum antibiotic and antifungal treatment, and a radiological response was observed.

Pertinent macroscopic findings at autopsy included pseudomembranous colitis involving the entire colon without evidence of perforation, ascites and pleural effusions, and haemorrhagic cystitis (positive for BK virus by PCR). Histological evaluation revealed no evidence of viral cytopathic effect, and bacterial, fungal and viral cultures from liver, lungs and spleen were all negative. There was no definite evidence of GVHD by histological evaluation of the skin, and there was no evidence of recurrent/residual leukaemia in any tissue examined. This was consistent with the pre-mortem skin biopsy performed on day 84, which showed no evidence of GVHD.

External examination of the liver revealed hepatomegaly (1625 g) with a smooth capsular surface. Sectioning of the liver revealed the usual soft brown parenchyma with multiple puckered, white-to-green lesions measuring that were distributed throughout the liver. Approximately half of these lesions were ultimately submitted for histological evaluation, and fungal and bacterial cultures of the liver lesions were also obtained but revealed no growth.

Microscopic examination of the liver parenchyma showed congestion with intact portal tracts and variable haemosiderin deposition. The lesional tissue consisted of irregular areas of localized, well-demarcated, non-bridging fibrosis with prominent brown-black pigment consistent with haemosiderin. Several of the foci contained granulomatous inflammation with aggregates of multinucleated giant cells and patchy lymphocytic inflammation. A modified Kinyoun stain revealed no evidence of acid-fast bacteria, but Mahan's methenamine silver and periodicacid–Schiff (PAS) stains for fungus showed rare aggregates of atypical-appearing intracellular organisms within the foci of granulomatous inflammation (Fig. 1b–d), so the decision was made to submit the tissue for molecular microbiological studies.

## Investigations

### Special stains

Formalin-fixed, paraffin-embedded blocks containing the organisms were stained with methenamine silver, PAS, modified Kinyoun, and FITE stains following standard histochemical protocols. The Fite's stain is a special histochemical reaction used to visualize acid fast organisms such as mycobacterium leprae and Nocardia.

## DNA extraction

The area of formalin-fixed, paraffin-embedded tissue that contained organisms was submitted *en bloc* for additional studies following macrodissection to enrich for organisms. The sample was deparaffinized in xylene prior to DNA extraction using a High Pure PCR Template preparation kit (Roche Diagnostics) according to the manufacturer's instructions.

## Molecular analysis

Universal bacterial and fungal PCR analyses targeting the 16S rRNA gene, 28S rRNA gene and internal transcribed spacer regions were performed in duplicate according to previously described protocols (Salipante *et al.*, 2012).

## Diagnosis

A 16S rRNA gene consensus length sequence was detected and demonstrated 100 % identity to *R.fascians* with GenBank accession no. X81930 (ATCC 12974^T^); no fungal DNA was detected.

## Discussion

The absence of other bacterial or fungal DNA in the setting of histologically visible organisms argues against the presence of *R.fascians* as a contaminant and raises the consideration that *R. fascians* represents a human pathogen for the immunocompromised. Whether it represented the sole infectious agent responsible for the liver lesions or a partially treated co-infection is impossible to determine, but our finding continues to reinforce the importance of molecular techniques in associating organisms with sites of infection and optimizing treatment of infectious diseases.

The finding of multiple fibrotic liver lesions was consistent with the report of numerous miliary lesions seen on CT scans performed at an outside hospital (reportedly decreased in size with treatment for presumed fungal infection). Most of these lesions consisted of well-demarcated sclerotic foci of fibrosis with two of the submitted lesions containing granulomatous inflammation that contained a small aggregate of atypical methenamine silver- and PAS-positive intracellular filamentous/hyphae forms possibly representing either filamentous bacteria or fungal hyphae. PCR studies for bacteria and fungi performed on this tissue revealed the presence of *R.fascians*. This organism (a member of the family *Nocardiaceae*) is a known plant pathogen that typically causes leafy gall disease; no cases of human infection have been reported. The related species *R.equi* has been associated with infections in immunocompromised patients; however, a review of the 18S (fungal) and 16S (bacterial) rRNA gene sequencing assays performed on an enriched specimen of the organisms showed only *R. fascians*. The presence of sclerotic foci and granulomas with thick fibrotic capsules may represent a partially treated infection, but these lesions were not considered to cause her death.

The most common complications of HCT are GVHD and infections with viral, bacterial and fungal agents (Horowitz & Bortin, 1990;Arnaout *et al.*, 2014). Prior to engraftment, patients who have received either a myeloablative or reduced intensity conditioning regimens are immunocompromised with a high risk of infection (Young *et al.*, 2016), reportedly 47–32 % in allogeneic marrow transplant patients. Our patient suffered the common post-HCT complications with both skin and gut GVHD and multiple infections including infection by an agent not previously known to cause human disease.

## Summary

This case highlights how unusual pathogens may cause infections in immunocompromised patients. We detected the presence of DNA with 100 % sequence identity for *R. fascians* in the liver of an immunosuppressed patient, a finding that suggests it may be a human pathogen. Our finding continues to reinforce the importance of molecular techniques in associating organisms with sites of infection and optimizing treatment of infectious diseases.
